# Dietary Fiber, Carbohydrate Quality and Quantity, and Mortality Risk of Individuals with Diabetes Mellitus

**DOI:** 10.1371/journal.pone.0043127

**Published:** 2012-08-23

**Authors:** Koert N. J. Burger, Joline W. J. Beulens, Yvonne T. van der Schouw, Ivonne Sluijs, Annemieke M. W. Spijkerman, Diewertje Sluik, Heiner Boeing, Rudolf Kaaks, Birgit Teucher, Claus Dethlefsen, Kim Overvad, Anne Tjønneland, Cecilie Kyrø, Aurelio Barricarte, Benedetta Bendinelli, Vittorio Krogh, Rosario Tumino, Carlotta Sacerdote, Amalia Mattiello, Peter M. Nilsson, Marju Orho-Melander, Olov Rolandsson, José María Huerta, Francesca Crowe, Naomi Allen, Ute Nöthlings

**Affiliations:** 1 Julius Center for Health Sciences and Primary Care, University Medical Center, Utrecht, The Netherlands; 2 Center for Prevention and Health Services Research, National Institute for Public Health and the Environment, Bilthoven, The Netherlands; 3 Department of Epidemiology, German Institute of Human Nutrition Potsdam-Rehbrücke, Nuthetal, Germany; 4 German Cancer Research Center, Heidelberg, Germany; 5 Department of Cardiology, Center for Cardiovascular Research, Aalborg Hospital, Aarhus University Hospital, Aalborg, Denmark; 6 Department of Epidemiology, School of Public Health, Aarhus University, Aarhus, Denmark; 7 Danish Cancer Society Research Center, Copenhagen, Denmark; 8 Navarre Public Health Institute, Pamplona, and Consortium for Biomedical Research in Epidemiology and Public Health (CIBER Epidemiología y Salud Pública-CIBERESP),Pamplona, Spain; 9 Molecular and Nutritional Epidemiology Unit, Istituto per lo Studio e la Prevenzione Oncologica, Florence, Italy; 10 Nutritional Epidemiology Unit, IRCCS Istituto Nazionale Tumori, Milan, Italy; 11 Cancer Registry and Histopathology Unit, “Civile M. P. Arezzo” Hospital, Ragusa, Italy; 12 Center for Cancer Prevention (Piedmont), and Human Genetic Foundation, Turin, Italy; 13 Department of Clinical and Experimental Medicine, Federico II University, Naples, Italy; 14 Department of Clinical Sciences, Lund University Hospital, Malmö, Sweden; 15 Department of Public Health and Clinical Medicine, Umeå University, Umeå, Sweden; 16 Department of Epidemiology, Murcia Regional Health Authority, and CIBER Epidemiología y Salud Pública (CIBERESP), Murcia, Spain; 17 Cancer Epidemiology Unit, University of Oxford, Oxford, United Kingdom; 18 Epidemiology Section, Institute for Experimental Medicine, Christian-Albrechts-University of Kiel, Kiel, Germany; German Diabetes Center, Leibniz Center for Diabetes Research at Heinrich Heine University Duesseldorf, Germany

## Abstract

**Background:**

Dietary fiber, carbohydrate quality and quantity are associated with mortality risk in the general population. Whether this is also the case among diabetes patients is unknown.

**Objective:**

To assess the associations of dietary fiber, glycemic load, glycemic index, carbohydrate, sugar, and starch intake with mortality risk in individuals with diabetes.

**Methods:**

This study was a prospective cohort study among 6,192 individuals with confirmed diabetes mellitus (mean age of 57.4 years, and median diabetes duration of 4.4 years at baseline) from the European Prospective Investigation into Cancer and Nutrition (EPIC). Dietary intake was assessed at baseline (1992–2000) with validated dietary questionnaires. Cox proportional hazards analysis was performed to estimate hazard ratios (HRs) for all-cause and cardiovascular mortality, while adjusting for CVD-related, diabetes-related, and nutritional factors.

**Results:**

During a median follow-up of 9.2 y, 791 deaths were recorded, 306 due to CVD. Dietary fiber was inversely associated with all-cause mortality risk (adjusted HR per SD increase, 0.83 [95% CI, 0.75–0.91]) and CVD mortality risk (0.76[0.64–0.89]). No significant associations were observed for glycemic load, glycemic index, carbohydrate, sugar, or starch. Glycemic load (1.42[1.07–1.88]), carbohydrate (1.67[1.18–2.37]) and sugar intake (1.53[1.12–2.09]) were associated with an increased total mortality risk among normal weight individuals (BMI≤25 kg/m^2^; 22% of study population) but not among overweight individuals (*P* interaction≤0.04). These associations became stronger after exclusion of energy misreporters.

**Conclusions:**

High fiber intake was associated with a decreased mortality risk. High glycemic load, carbohydrate and sugar intake were associated with an increased mortality risk in normal weight individuals with diabetes.

## Introduction

The total number of adults with diabetes is expected to rise to 439 million, 6.4% of the world adult population in 2030 [1]. Diabetes patients have a more than twofold increased risk of both micro- and macrovascular complications leading to high morbidity and mortality. Cardiovascular disease CVD is the primary cause for the decreased life expectancy of patients with diabetes [2], [3].

Morbidity and mortality of patients with diabetes are associated with the degree of hyperglycemia [4]. Therapeutic lifestyle interventions including dietary modification have the potential to improve glycemic control with little risk of hypoglycemia [5]–[7]. The glycemic index (GI) is an indicator of the average quality of the carbohydrates consumed in terms of glycemic response [8]. Glycemic load (GL) is calculated by multiplying the GI of a food with its carbohydrate content and represents both quality and quantity [9], [10]. Several studies have shown that high dietary GL or GI were associated with an increased risk of diabetes and CVD, especially among overweight women [11]–[16]. Overweight and obesity are usually accompanied by insulin resistance which exacerbates the postprandial glucose response and may amplify the increased CVD risk of high dietary GL and GI. Because insulin resistance is a key characteristic of type 2 diabetes [17], GI and GL may be important risk factors for CVD in diabetes patients.

Several randomized controlled trials have shown that low-GI diets improved glycemic control in diabetes patients, as measured by glycated hemoglobin [18]–[20]. However, the relation of carbohydrate quantity and quality with CVD among diabetes patients is largely unknown. One study examined GL and GI in relation to CVD risk in a small subgroup of type 2 diabetes patients [21]. High GL and GI were associated with an increased coronary heart disease risk among individuals without diabetes, but no significant associations were observed for patients with type 2 diabetes. Among US women with type 2 diabetes, whole-grain and bran intakes were associated with reduced all-cause and CVD mortality [22]. In line with these findings, the dietary recommendations for CVD prevention in patients with diabetes promote ample intake of dietary fiber, but do not provide specific recommendations on carbohydrate quality or quantity [23]. A recent statement by the American Diabetes Association, however, advocates that glycemic index may provide a modest benefit in the medical care of diabetes patients [24]. Therefore, the aim of the current study is to investigate whether dietary fiber intake, carbohydrate quality and quantity are associated with all-cause and CVD mortality risk in a large European cohort of men and women with confirmed diabetes mellitus. Because previous studies in the general population have shown that sex, BMI and smoking status may modify the association of dietary GI and GL with CVD [11], [13], [14], [25], we aimed to investigate effect modification by these factors. Finally, because previous studies on GI and GL in relation to weight gain or incidence of type 2 diabetes showed that energy misreporting affected the strength of the associations [15], [26], we also analyzed the effect of energy misreporting.

## Methods

### Ethics Statement

The study complied with the Declaration of Helsinki and was approved by the ethical review boards of the International Agency for Research on Cancer and from all local institutions. All participants gave written informed consent prior to inclusion. The full names of the 15 EPIC centres: ISPO Cancer Prevention and Research Institute, Florence, Italy; Fondazione IRCCS Istituto Nazionale dei Tumori, Milan, Italy; U.O.S. Registro Tumori e U.O.C. Anatomia Patologica, Ospedale “Civile M.P.Arezzo” ASP 7, Ragusa, Italy; University of Torino and HuGEF Foundation, Turin, Italy; Universita di Napoli, Federico II, Naples, Italy; Instituto de Salud Pública Gobierno de Navarra, Pamplona, Spain; Subdirección de Salud Pública de Gipuzkoa, Gobierno Vasco, San Sebastian, Spain; Centre for Nutrition and Health, National Institute of Public Health and the Environment (RIVM), Bilthoven, The Netherlands; Julius Center for Health Sciences and Primary Care, Dept of Epidemiology, University of Utrecht, The Netherlands; German Cancer Research Centre, Heidelberg, Germany; Deutsches Institut für Ernährungsforschung, Potsdam-Rehbrücke, Germany; Malmö University Hospital, Lund University, Malmö, Sweden; Dept of Public Health and Clinical Medicine, Umeå University, Umeå, Sweden; Dept of Epidemiology, Institute of Public Health, Aarhus University, Aarhus, Denmark; The Danish Cancer Society, Institute of Cancer Epidemiology, Copenhagen, Denmark

### Population

This study is nested within EPIC, an ongoing multi-center cohort study of 519,978 men and women from ten European countries [27]. Participants were 35 to 70 years at enrolment between 1992 and 2000 and mostly recruited from the general population.

Using additional data on diabetes diagnosis at baseline provided by fifteen EPIC study centers from six European countries (Denmark, Germany, Italy, The Netherlands, Spain, Sweden), allowed the formation of a sub-cohort of individuals with a confirmed diagnosis of diabetes mellitus. Self-reports of a diabetes diagnosis at baseline were confirmed by contact to a medical practitioner (in 43%), repeated self-report during follow-up (in 27%), the reported use of diabetes-related medication, e.g. use of insulin or oral hypoglycemic agents (23%), a baseline HbA1c above 6% (in 7%; measured in Malmö only), both by contact to a medical practitioner and the reported use of diabetes-related medication (<1%), or by the use of regional diabetes registers (<1%). Of the initial 7,048 self-reports in the participating EPIC centers, 5,542 participants were confirmed to have had diabetes at baseline. Subsequently, 870 additional cases were included because they turned out to have been prevalent diabetes cases. This led to a cohort comprising 6,412 individuals with confirmed diabetes at study entry [28]. After exclusion of participants with missing dietary information (n = 42), participants in the highest or lowest 1% of the ratio of energy intake to estimated energy requirement (n = 177) [14], [29]–[31], and deceased participants with missing date of death (n = 1), the analytical sample comprised of 6,192 participants with diabetes mellitus.

### Baseline Measurements

#### Dietary Assessment

In EPIC, dietary intake during the previous year was assessed at baseline by means of self-administered country-specific questionnaires [32], either quantitative dietary questionnaires with individual portion sizes (in France, Spain, the Netherlands, Germany and Italy, except Naples) or semi-quantitative food frequency questionnaires (FFQ) (in Denmark, Naples (Italy), Sweden, and the UK), that were developed and validated locally [33]–[41]. Correlation coefficients for the relative validity for carbohydrate measured with FFQ varied from 0.40 in Denmark to 0.84 in Spain for men, and from 0.46 in Malmö (Sweden) to 0.78 in Spain for women [34]. Correlation coefficients for dietary fiber ranged from 0.33 (Oxford, UK) to 0.74 (women in the Netherlands). Dietary GI and GL measured with FFQ have been validated against twelve 24 h dietary recalls in the Dutch contribution to the EPIC cohort, and Spearman correlations were 0.62 for GI and 0.60 for GL [42]. Detailed descriptions on the usual dietary intake have been described elsewhere [43]. The GI of foods was obtained from a GI database specially developed for this purpose, using glucose as the reference, and GI values were assigned to items reported in the dietary questionnaires in a standardized manner as described in detail elsewhere [44]. In brief, foods reported in the dietary questionnaires were judged on the basis of the GI value of the food while considering aspects of the food that might influence GI (e.g., cooking method, preservation method, sugar content and country-specific types of food). GI values obtained from the Foster-Powel table [45], British values [46], and internet updates (http://www.glycemicindex.com) were then assigned to individual food items. No value was assigned for food items that contained no or a negligible amount of carbohydrate or foods that do not increase blood glucose levels (chiefly meat and fish, fats, eggs). GI values were updated in 2009, using the recently published table by Atkinson et al [47]. Average dietary GL was calculated by adding up the products of digestible carbohydrate for each food (g per day) and its GI. Average dietary GI was calculated as GL, but by dividing by the total amount of digestible carbohydrate in one day [9], [10]. Intakes of nutrients were adjusted for total energy intake by means of the regression residual method [48]. BMR was estimated with the use of the Schofield equations, and used as a measure of individual energy requirement. Participants with an energy intake compared with energy requirement of <1.14 were defined as under-reporters, whereas those with an energy intake compared with energy requirement of >2.40 were classified as over-reporters [31]. The remaining participants were defined as normal energy reporters.

#### Measurement of Nondietary Factors

All participants underwent baseline anthropometric and blood pressure measurements. Further lifestyle- and health-related variables were collected at baseline using a general questionnaire. Physical activity level was indexed into four categories (inactive, moderately inactive, moderately active, active) [49]. Diabetes duration was calculated from the date of diagnosis supplied by the medical practitioner in the confirmation process (if available) or by self-reported age at diagnosis. Insulin use was defined by self-reported diabetes related medication at baseline. At baseline, all participants donated a non-fasting blood sample. Blood samples were stored at −80°C or −196°C. For all centers except Potsdam (Germany), HbA1c and hemoglobin were measured on an auto-analyzer (LX20-Pro, Beckman-Coulter), using a turbidimetric immuno-inhibition method, and a colorimetric method at 410 nm, respectively. In EPIC-Potsdam, HbA1c was measured with the automatic ADVIA 1650 analyzer (Siemens Medical Solutions, Erlangen, Germany).

### Morbidity and Mortality Follow-up

Information on vital status, cause and date of death, were obtained by using follow-up mailings and subsequent inquiries to municipal registries, regional health departments, physicians, or hospitals (Germany), or by record linkages with local, regional, or central cancer registries, boards of health, or death indexes (other countries). Mortality data were coded following the 10^th^ revision of the International Classification of Diseases (ICD-10). CVD mortality (ICD-10 [I00-I99]), combining primary and secondary cause of death, was used as a secondary outcome measure.

### Statistical Analysis

Missing values were present in glycated hemoglobin (31.5%), duration of diabetes, smoking duration, physical activity, and WHR (<6.6%). Because missing values seldom occur completely at random, removing patients with missing values from the analysis may yield biased results [50], [51]. Therefore, to reduce bias, missing scores were imputed by single linear regression modeling (Statistical Package for Social Sciences (SPSS), Missing Value Analysis procedure). Associations between dietary factors and mortality risk were estimated using multivariate Cox proportional hazard models stratified by sex and country, with age as the primary time variable. The proportional hazard assumption was checked visually using log-minus-log plots with no deviations detected. HRs were expressed per SD of intake: 6.4 g/day for fiber, 22.0 g/day for GL, 3.9 for GI, 35.4 g/day for carbohydrate, 31.0 g/day for sugar, and 31.6 g/day for starch. Model selection was based on a pre-selection of potential confounders and testing these factors in a univariable model to select the factors that influenced the relation between determinant and outcome most. We subsequently adjusted for the selected factors in 4 steps. In the first model (model M1) HRs were not adjusted. The second model included CVD-related risk factors: smoking (categorical), smoking duration (continuous), education (categorical), BMI (categorical), WHR (continuous), physical activity (categorical), alcohol intake (categorical), menopausal status (pre, post), and HRT (hormone replacement therapy) use (model M2). In a third model (M3) factors associated with severity of diabetes were included; diabetes duration (continuous), insulin use (yes, no), and glycated hemoglobin level (continuous). The final model (model M4) was additionally adjusted for the following dietary factors; total energy (continuous), vitamin C, and saturated, monounsaturated, and polyunsaturated fat (continuous). Final models for GL, GI, carbohydrate, sugar, and starch were additionally adjusted for dietary fiber (continuous), while final models for GI and dietary fiber were also adjusted for carbohydrates (continuous). In separate analyses, BMI, WHR and glycated hemoglobin, potential intermediates, were removed from the full model. Nonlinear associations were explored by inclusion of quadratic terms in the final model (all non-significant; Wald p-values >0.15) and by modeling the nutrients in quartiles, but no evidence of non-linearity was detected. To study whether sex, BMI, smoking status, or fiber intake (for GL only) modified the association between exposure variable and mortality risk, interaction terms were added to the fourth model for each variable separately, and tested for significance with a likelihood ratio test. All analyses were carried out on the complete study population, as well as, in a sensitivity analysis, after exclusion of energy mis-reporters (N = 2354; 2301 under-reporters, and 53 over-reporters). In additional sensitivity analyses (model M4), we restricted the study population to participants being diagnosed with diabetes at age 40 or older (N = 4901), or reporting not to use insulin (N = 4809), in an attempt to exclude patients with type 1 diabetes. We also excluded participants with (potential) co-morbidities at baseline, by leaving out cases occurring in the first two years of follow-up, or by omitting participants with a history of heart disease, stroke, or cancer, at baseline. Finally, we adjusted for use of oral hypoglycemic medication, hypertension and hyperlipidemia and, in the analyses of dietary fiber, for magnesium intake. Data were analyzed with SAS (version 9.2; SAS Institute Inc., Cary, NC). A two-sided p-value of 0.05 was considered statistically significant. Heterogeneity across study countries was evaluated using the DerSimonian and Laird random effects model (metan procedure, STATA 11, StataCorp, Texas, USA).

## Results

Median diabetes duration was 4.4 years and 22% of participants reported to use insulin (Table 1). During 56,969 person-years of follow-up, 791 deaths (533 men, 258 women) were recorded, 306 died of CVD (215 men), 163 died of cancer (103 men), and 118 died of other known causes (74 men). Dietary GL correlated strongly with carbohydrate intake (Pearson r = 0.93), while GI (r = 0.12), sugar (r = 0.58), and starch (r = 0.56) only showed weak to moderate correlations with carbohydrate intake. Weak to moderate correlations were also observed between dietary fiber intake and GL (r = 0.33), GI (r = −0.04), carbohydrate (r = 0.38), sugar (r = 0.07), or starch (r = 0.37). Over the quartiles of dietary fiber intake, the percentage of individuals using insulin, and intakes of carbohydrates and vitamin C increased, whereas intakes of monounsaturated fat, saturated fat and alcohol, and percentages of men, smokers and physically inactive individuals decreased (Table S1). Over the quartiles of carbohydrate intake, mean intake of dietary fiber and vitamin C increased, whereas median duration of diabetes, intakes of fat and alcohol, and percentages of men, and smokers decreased. Dietary fiber, GL, carbohydrate, and starch were inversely associated with all-cause mortality risk controlling for country, sex and age (Table 2, model M1). This inverse association persisted after further adjustment for CVD-related and diabetes-related risk factors (models M2 and M3). After adjustment for nutritional factors, the inverse associations of GL, carbohydrate, and starch with all-cause mortality risk attenuated, and only high intake of dietary fiber remained associated with a reduced all-cause mortality risk (model M4; HR per SD increase in dietary fiber intake: 0.83; 95% CI, 0.75–0.91). No associations were observed for dietary GI or sugar intake.

**Table 1:** Baseline characteristics of the study population.

		All participants	Normal energy reporters
N		6192	3838
Energy under-reporters (n, %)		2301 (37.2)	0 (0)
Energy over-reporters (n, %)		53 (0.9)	0 (0)
Male sex (n, %)		3355 (54.2)	2139 (55.7)
Glycemic Load (g/d)		116.9±22.0*	117.5±22.0
Glycemic Index		55.2±3.9	55.2±3.9
Age (yrs)		57.4±6.7	57.6±6.8
BMI (kg/m^2^)		28.8±4.9	27.9±4.6
WHR		0.92±0.09	0.92±0.09
Physical Activity (%)			
Inactive		30.6	29.4
Mod Inactive		32.4	32.6
Mod Active		20.0	19.7
Active		17.0	18.4
Education (%)			
Low		45.8	44.2
Middle		37.9	38.1
High		16.4	17.7
Smoking (%)			
Never		39.2	38.4
Former		35.7	35.1
Current		25.1	26.6
Systolic blood pressure (mm Hg)		145.2±21.0	144.8±21.2
Diastolic blood pressure (mm Hg)		85.4±11.0	84.7±10.9
Hypertension (%)		50.9	46.5
Hypercholesterolemia (%)		42.6	40.1
HbA1c (% of total hemoglobin)		8.1±1.9	8.0±1.9
Menopausal status (% post)		77.9	77.5
OC use (%)		1.7	1.8
HRT use (%)		13.8	14.0
Age at diabetes diagnosis (yrs)		50.1±9.8	50.2±10.1
Duration of diabetes (yrs)		4.4 (1.8–9.7)**	4.4 (1.9–9.2)
Insulin use (%)		22.3	23.1
Use of glucose-lowering drugs (%)		82.1	81.9
Nutrients (daily intake)†			
Total Energy (kcal)		2047±639	2364±543
Carbohydrate (g)		211.5±35.4	212.5±35.3
Sugar (g)		84.5±31.0	85.5±31.2
Starch (g)		121.8±31.6	122.4±31.8
Protein (g)		89.2±16.0	89.3±15.5
Total Fat (g)		76.3±13.5	76.3±13.3
Polyunsaturated Fat (g)		13.2±4.6	13.1±4.6
Monounsaturated Fat (g)		28.0±7.4	28.1±7.2
Saturated Fat (g)		28.9±7.3	29.0±7.3
Fiber (g)		23.5±6.4	23.8±6.4
Alcohol (g)		5.7 (0.6–20.7)	8.3 (1.1–25.9)
Vitamin C (mg)		114.3±55.6	115.6±55.4

* Mean ± SD (all such values);

** Median (IQR; all such values); normal energy reporters were defined as energy intake compared to basal metabolic rate of ≥1.14 and ≤2.40;

† nutritional variables were adjusted for total energy intake, except alcohol and energy. BMI = body mass index; WHR = waist-to-hip ratio; OC = oral contraceptives; HRT = hormone replacement therapy.

**Table 2:** Dietary fiber, glycemic load, glycemic index, carbohydrate, sugar, and starch, and all-cause mortality risk among 6,192 individuals with diabetes mellitus*.

Model	Fiber°	Glycemic load	Glycemic index°	Carbohydrate	Sugar∥	Starch∥
M1: crude	0.81 (0.75–0.87)	0.87 (0.81–0.94)	0.97 (0.90–1.05)	0.87 (0.81–0.94)	1.03 (0.96–1.11)	0.80 (0.74–0.87)
M2: M1+CVD-related risk factors†	0.87 (0.80–0.94)	0.90 (0.83–0.98)	0.96 (0.89–1.03)	0.91 (0.84–0.99)	1.03 (0.96–1.11)	0.84 (0.77–0.92)
M3: M2+diabetes risk factors‡	0.84 (0.78–0.92)	0.92 (0.84–1.00)	0.96 (0.89–1.04)	0.92 (0.85–1.00)	1.06 (0.98–1.14)	0.83 (0.76–0.91)
M4: M3+dietary intake§	0.83 (0.75–0.91)	1.01 (0.89–1.14)	0.99 (0.91–1.07)	1.03 (0.89–1.19)	1.04 (0.91–1.19)	0.93 (0.80–1.07)

* Adjusted Hazard Ratios (with 95% CI) per SD of fiber (6.4), GL (22.0), GI (3.9), carbohydrate (35.4), sugar (31.0), and starch (31.6). Age was used as the primary time variable, and all models were stratified on sex and country.

† Adjusted for smoking (never, past, current with ≤10 cig/d, current with 10–20 cig/d, current ≥20 cig/d), smoking duration (continuous), education (low, middle, high), BMI (<18.5, 18.5–25, 25–30, ≥30 kg/m^2^), WHR (continuous), physical activity (inactive, moderately inactive, moderately active, active), menopausal status (pre, post), HRT use (ever, never), and alcohol (≤10, 10–25, 25–50, >50 g/day).

‡ Adjusted for diabetes duration (continuous), insulin use (yes, no), HbA1c (continuous), and covariates from footnote†.

§ Adjusted for total energy (continuous), and energy-adjusted nutrients (all continuous), vitamin C, and saturated, monounsaturated, and polyunsaturated fat, and covariates from footnote‡. Models M4 for GL, GI, carbohydrate, sugar, and starch were also adjusted for energy-adjusted fiber intake (continuous).

° Model M4 for GI and fiber were also adjusted for energy-adjusted carbohydrate intake.

∥ Model M4 for sugar and starch, contained both sugar and starch.

Among the 791 death cases (533 men, 258 women), 306 (215 men) died of CVD, 163 (103 men) died of cancer, and 118 (74 men) died of other known causes. GL = dietary glycemic load; GI = dietary glycemic index; M = model; WHR = waist-to-hip ratio; HbA1c = glycated hemoglobin (% of total hemoglobin).

We observed interactions of GL, carbohydrate, and sugar, with BMI (Table 3, model M4; *P*interaction<0.04). Subgroup analyses differentiating between normal weight and overweight individuals (BMI below and above 25 kg/m^2^), showed positive associations of GL [1.42; CI, 1.07–1.88], carbohydrate [1.67; CI, 1.18–2.37], and sugar [1.53; CI, 1.12–2.09] in the normal weight category, but slightly negative and non-significant associations among overweight individuals.

**Table 3:** Dietary fiber, glycemic load, glycemic index, carbohydrate, sugar, and starch, and all-cause mortality risk among 6,192 individuals with diabetes mellitus, before and after exclusion of energy mis-reporters, as well as in BMI subgroups*.

	Subgroups	All participants†	cases	Normal energy reporters	cases
Fiber	Overall	0.83 (0.75–0.91)	791	0.84 (0.74–0.95)	498
p = 0.87 (0.72)**	BMI≤25 kg/m^2^	0.71 (0.58–0.87)	183	0.70 (0.55–0.89)	139
	BMI>25 kg/m^2^	0.86 (0.77–0.96)	608	0.89 (0.77–1.04)	359
Glycemic load	Overall	1.01 (0.89–1.14)	791	1.15 (0.99–1.34)	498
p = 0.04 (0.13)	BMI≤25 kg/m^2^	1.42 (1.07–1.88)	183	1.74 (1.23–2.46)	139
	BMI>25 kg/m^2^	0.93 (0.81–1.06)	608	1.03 (0.87–1.23)	359
Glycemic index	Overall	0.99 (0.91–1.07)	791	1.03 (0.92–1.14)	498
p = 0.79 (0.88)	BMI≤25 kg/m^2^	1.00 (0.84–1.19)	183	1.06 (0.86–1.32)	139
	BMI>25 kg/m^2^	0.98 (0.90–1.08)	608	1.02 (0.90–1.15)	359
Carbohydrate	Overall	1.03 (0.89–1.19)	791	1.18 (0.98–1.43)	498
p = 0.02 (0.15)	BMI≤25 kg/m^2^	1.67 (1.18–2.37)	183	2.04 (1.34–3.10)	139
	BMI>25 kg/m^2^	0.92 (0.78–1.09)	608	1.03 (0.83–1.27)	359
Sugar	Overall	1.04 (0.91–1.19)	791	1.13 (0.96–1.34)	498
p = 0.01 (0.09)	BMI≤25 kg/m^2^	1.53 (1.12–2.09)	183	1.76 (1.21–2.56)	139
	BMI>25 kg/m^2^	0.96 (0.83–1.11)	608	1.01 (0.84–1.23)	359
Starch	Overall	0.93 (0.80–1.07)	791	1.08 (0.90–1.31)	498
p = 0.77 (1.00)	BMI≤25 kg/m^2^	1.29 (0.92–1.80)	183	1.60 (1.07–2.39)	139
	BMI>25 kg/m^2^	0.86 (0.73–1.02)	608	0.97 (0.78–1.21)	359

* Adjusted Hazard Ratios (with 95% CI) per SD of fiber (6.4), GL (22.0), GI (3.9), carbohydrate (35.4), sugar (31.0), and starch (31.6). Full models M4 (see footnotes of Table 2). Normal energy reporters (n = 3838) were defined as energy intake compared to basal metabolic rate of ≥1.14 and ≤2.40.

** Interaction analysis based on continuous interaction terms between respective dietary factors and BMI, for all participants, or normal energy reporters only (in brackets).

† Overall Hazard ratios identical to those in Table 2.

Among the 791 death cases (533 men, 258 women), 306 (215 men) died of CVD, 163 (103 men) died of cancer, and 118 (74 men) died of other known causes. There were 498 cases (342 men) among normal energy reporters, 181 (129 men) died of CVD, 95 (60 men) died of cancer, and 75 (50 men) died of other known causes. Of the individuals with diabetes, 22% (27% of normal energy reporters) fell into the normal weight category (BMI≤25 kg/m^2^).

In total, 37.2% of participants were classified as energy under-reporters and 0.9% as energy over-reporters (Table 1). Energy under-reporters were more often female, physically less active, and had a higher BMI compared with normal energy reporters; the opposite was observed for energy over-reporters (see Table S2). After exclusion of energy under- and over-reporters, high intakes of GL [1.15; CI, 0.99–1.34], and carbohydrate [1.18; CI, 0.98–1.43], tended to be associated with an increased all-cause mortality risk (Table 3). Exclusion of energy under- and over-reporters did not affect the inverse association between fiber intake and mortality risk (HR: 0.84; CI, 0.74–0.95). Restricting the analysis to normal energy reporters augmented the associations of GL [1.74; CI, 1.23–2.46], carbohydrate [2.04; CI, 1.34–3.10], sugar [1.76; CI, 1.21–2.56], and starch [1.60; CI, 1.07–2.39] with mortality risk in normal weight individuals. Again, no significant associations were found in overweight individuals. No significant interaction was observed between sex or smoking status and GI, GL, fiber, or carbohydrate, or between GL and fiber intake (*P*interaction>0.17).

Sensitivity analyses, replacing the categorical variable for BMI by a continuous variable, removing BMI, WHR and glycated hemoglobin from the multivariate models, adjusting for oral hypoglycemic medication, hypertension and hyperlipidemia, or adjusting fiber analyses for magnesium intake, did not appreciably affect the results. Associations also did not change by excluding prevalent CVD and cancer, or by excluding cases occurring in the first two years of follow-up. Similarly, restricting the analysis to participants with an age at diabetes diagnosis above 40 years (N = 4901), or to participants not using insulin (N = 4809), did not affect our conclusions. We did not observe significant heterogeneity between country-specific effect estimates of all cause mortality risk for any of the determinants (I^2^<41%; Figure S1).

Separate analyses of CVD mortality risk (Table 4) showed similar results, with no associations for GL, GI, or carbohydrate (subtype), and an inverse association of dietary fiber (HR: 0.76; CI, 0.64–0.89). An inverse association was also observed for dietary fiber in analyses of mortality risk due to cancer and other causes, (HR: 0.82; CI, 0.66–1.02 and HR: 0.65; CI, 0.50–0.85).

**Table 4:** Dietary fiber, glycemic load, glycemic index, carbohydrate, sugar, and starch, and CVD mortality risk among 6,192 individuals with diabetes mellitus, before and after exclusion of energy mis-reporters, as well as in BMI subgroups*.

	Subgroups	All participants	cases	Normal energy reporters	cases
Fiber	Overall	0.76 (0.64–0.89)	306	0.76 (0.62–0.94)	181
p = 0.10 (0.52)**	BMI≤25 kg/m^2^	0.67 (0.48–0.95)	70	0.54 (0.34–0.86)	48
	BMI>25 kg/m^2^	0.75 (0.62–0.90)	236	0.78 (0.60–1.01)	133
Glycemic load	Overall	0.95 (0.78–1.15)	306	1.03 (0.80–1.32)	181
p = 0.03 (0.32)	BMI≤25 kg/m^2^	1.15 (0.72–1.83)	70	1.11 (0.61–2.05)	48
	BMI>25 kg/m^2^	0.93 (0.75–1.16)	236	1.03 (0.77–1.39)	133
Glycemic index	Overall	0.96 (0.85–1.10)	306	0.96 (0.81–1.14)	181
p = 0.86 (0.72)	BMI≤25 kg/m^2^	0.83 (0.63–1.11)	70	0.88 (0.60–1.28)	48
	BMI>25 kg/m^2^	1.01 (0.87–1.17)	236	1.01 (0.83–1.23)	133
Carbohydrate	Overall	0.97 (0.77–1.23)	306	1.10 (0.80–1.50)	181
p = 0.02 (0.22)	BMI≤25 kg/m^2^	1.68 (0.93–3.04)	70	1.56 (0.74–3.31)	48
	BMI>25 kg/m^2^	0.89 (0.69–1.16)	236	1.03 (0.72–1.47)	133
Sugar	Overall	0.96 (0.78–1.18)	306	1.04 (0.79–1.35)	181
p = 0.03 (0.10)	BMI≤25 kg/m^2^	1.52 (0.89–2.60)	70	1.42 (0.73–2.76)	48
	BMI>25 kg/m^2^	0.90 (0.72–1.12)	236	0.99 (0.73–1.33)	133
Starch	Overall	0.89 (0.71–1.12)	306	0.99 (0.73–1.34)	181
p = 0.36 (0.90)	BMI≤25 kg/m^2^	1.27 (0.71–2.26)	70	1.08 (0.52–2.27)	48
	BMI>25 kg/m^2^	0.89 (0.69–1.14)	236	1.02 (0.72–1.44)	133

* Adjusted Hazard Ratios (with 95% CI) per SD of fiber (6.4), GL (22.0), GI (3.9), carbohydrate (35.4), sugar (31.0), and starch (31.6). Full models M4 (see footnotes of Table 2). Normal energy reporters (n = 3838) were defined as energy intake compared to basal metabolic rate of ≥1.14 and ≤2.40.

** Interaction p-values are based on analysis of all participants or normal energy reporters only (in brackets), and using continuous interaction terms for BMI.

Of the individuals with diabetes, 22% (27% of normal energy reporters) fell into the normal weight category (BMI≤25 kg/m^2^). There were 70 CVD deaths (46 men) in the normal weight category, and 236 (169 men) in the overweight category, 48 (30 men) and 133 (99 men) among normal energy reporters.

## Discussion

Our main finding is that in individuals with diabetes mellitus, higher dietary fiber intake was associated with a reduced mortality risk. For the other exposures, GL, GI, carbohydrate, sugar, and starch, no statistically significant associations were observed in the complete study population. Diets with high GL, carbohydrate and sugar intake were associated with increased mortality risk among normal energy reporters and normal weight individuals, but not among overweight individuals.

The inverse association of dietary fiber with all-cause and CVD mortality risk is in line with previous findings in the general population [52], as well as in individuals with diabetes [22], [53]. A study performed among US women with type 2 diabetes showed that whole-grain and bran intakes were associated with reduced all-cause and CVD mortality [22]. An earlier study within EPIC of 10,449 participants with self-reported (unconfirmed) diabetes showed that intake of vegetables, legumes, and fruit were associated with reduced risks of all-cause and CVD mortality [53]. Although we could not differentiate subtypes of dietary fiber, the current data suggest that dietary fiber from these sources may have contributed to the reduced mortality risk.

We did not observe associations of GL and GI with mortality risk in analyses of the complete study population. However, positive associations of GL, total carbohydrate, and sugar, with mortality risk in individuals with diabetes were observed after restricting our analysis to normal weight individuals or, in a post hoc sensitivity analysis, after excluding energy mis-reporters. Note that dietary GL correlated strongly with total carbohydrate intake (Pearson r = 0.93). So far, one study examined GL and GI in relation to CVD risk in a sub-cohort of 1,378 US citizens with type 2 diabetes [21]. No associations of GL and GI with CVD were observed in Whites, while non-significant positive associations were found in African Americans. The relatively low GI and GL, the small sample size, the fact that energy mis-reporters were not excluded, and that interaction with BMI was not studied, may explain the different findings in this study.

In comparison with studies performed in the general population typically showing ∼25% energy misreporting [15], [26], a relatively high proportion of our diabetes patients misreported their energy intake. This can be explained by differences between the study populations. Energy misreporting is more pronounced in overweight or obese as compared to normal weight individuals [54], [55], and diabetes patients appear to underreport even more than their obese counterparts [56]. Energy under-reporters tend to specifically misreport foods that largely contribute to the GL and GI, such as sugars, cookies, milk products (relatively low intakes reported), and fruit and vegetables (relatively high intakes reported) [54]. Thus, consideration of energy misreporting may be particularly important studying health effects of carbohydrates in overweight individuals and diabetes patients. Previous studies on GI and GL in relation to weight gain or incidence of type 2 diabetes showed that energy misreporting affected the strength of the associations [15], [26], while the associations for dietary fiber were hardly influenced by excluding energy under-reporters [15]. Our results are in agreement with these results showing that misreporting affected associations for dietary GI and GL but not for dietary fiber. Probably due to the high prevalence of misreporting among patients with diabetes, the effects of energy misreporting are particularly strong in the current study, where associations of GL and carbohydrates (and its subtypes) with mortality risk only appeared after exclusion of energy mis-reporters. Indeed, positive associations of GL and carbohydrates with mortality risk would be attenuated in the presence of energy under-reporters that report too low carbohydrate and sugar intakes.

Dietary GL, carbohydrate and sugar intake were associated with an increased mortality risk in normal weight individuals only. These findings are not due to selective misreporting of obese individuals, since the same result was obtained after exclusion of energy mis-reporters among normal weight persons. Moreover, exclusion of type 1 diabetes patients based on insulin use or age at diabetes diagnosis, did not weaken the associations. After exclusion of insulin users, HRs for the association between carbohydrate intake and all-cause mortality risk were 2.20 [CI, 1.36–3.55] and 0.89 [CI, 0.74–1.08] in normal and overweight diabetes patients respectively. Similarly, for patients with an age at diabetes diagnosis over 40 years, the association between carbohydrate intake and all-cause mortality risk were 1.77 [CI, 1.15–2.73] and 0.90 [CI, 0.75–1.08] in normal and overweight diabetes patients respectively. Thus, our data identify a group of normal weight individuals with type 2 diabetes (22% of our diabetes patients) having a high susceptibility to dietary carbohydrates. Indeed, about 20% of Caucasian patients with type 2 diabetes have BMI<25 kg/m^2^ [57]. On average, normal weight patients were diagnosed with diabetes at a younger age, were more likely to use insulin, and less likely to have hypertension or hypercholesterolemia (Table S3). Obese and non-obese diabetes patients share the same CVD risk factors, and have a similar CVD risk [58]. However, non-obese diabetes patients have a more deficient insulin secretion and less peripheral insulin resistance as compared to obese diabetes patients indicating a different cause of hyperglycemia [59]. Because of a less efficient insulin response, non-obese diabetes patients may respond to high GL diets with more severe hyperglycemia and higher CVD risk. Our data suggest that high GL, carbohydrate, and sugar, are important risk factors for these non-obese diabetes patients.

In contrast to several studies performed in the general population [11]–[14], [16], [60], we did not find associations between GI and all-cause, CVD, or cancer (data not shown) mortality risk in patients with diabetes. This suggests that GI may be a more important risk factor in individuals without diabetes. GI may be a relatively less important risk factor in patients with diabetes where hyperglycemia is primarily due to peripheral insulin resistance (and deficient insulin secretion). Randomized trials have shown that low-GI and low-GL diets affect plasma concentrations of LDL-cholesterol, HDL-cholesterol, total cholesterol, triglycerides and markers of inflammation and thrombosis, as well as insulin resistance, in ways that would be expected to decrease CVD risk ([61]–[65]. Similarly, dietary fiber may reduce CVD risk by improving serum lipid profiles [66], postprandial absorption and insulin resistance [67], lowering blood pressure [68], or by its anti-inflammatory properties [69], [70].

An important strength of the current study is that diabetes status did not depend on self-report only, but was confirmed, minimizing misclassification. Moreover, our study population is a sub-cohort of EPIC, offering a multi-centric design across diverse countries with a large sample size and long follow-up time. However, certain limitations need to be addressed. This study relied on baseline information with respect to diabetes diagnosis, dietary intake, and use of medication because updated information at follow-up was not available. Although this could lead to misclassification due to changes during follow-up, our results for dietary fiber are in line with those by He et al [22] using updated information of dietary intake. Residual confounding cannot be excluded, but is made less likely by the large number of risk factors that we adjusted for. Misclassification of dietary exposure is a valid concern, in particular because dietary information was obtained through self-report. The FFQs have been validated (see Methods section) showing reasonable to good validity for most food groups as well as for dietary fiber, GL and GI [34]. Although these FFQs were not specifically designed to measure GL and GI, a validation study in the Dutch EPIC cohort (EPIC-NL) reported good agreement of the FFQ with 24 h dietary recalls for GL and GI [42]. An overall assessment of GI methodology within EPIC concluded that ranking of participants to their GL values is acceptable but the ranking according to GI should be used with care [71]. However, non-differential misclassification will only have attenuated our results. Finally, the relative validity for carbohydrate measured with FFQ was generally reported to be moderate to good in each country/centre that contributed to the EPIC cohort [34]. To identify energy mis-reporters, BMR was calculated using the Schofield equations, which may not be completely adequate for diabetic patients. Because BMR may be higher in diabetic patients [72], [73], we may have slightly underestimated the number of energy under-reporters. Moreover, energy misreporting was defined assuming an average physical activity level (PAL) of 1.55 typical for a sedentary lifestyle [31]. Thus, normal energy reporters with lower than average physical activity may have been misclassified as energy under-reporters. However, similar results were obtained when we assigned typical physical activity levels (PAL-values; 1.2, 1.5, 1.8, and 2.1) to the four categories of physical activity, and estimated individual energy requirement based on BMR as well as physical activity (data not shown). It is therefore unlikely that misclassification of energy reporting influences our results to a large extent. As in any observational study, our results could be influenced, at least in part, by differences in factors other than dietary fiber, GI or GL. We simultaneously controlled for a large range of potential confounding factors, but unmeasured confounding cannot be excluded. Fiber intake in particular is associated with health-seeking behaviours and the inverse association of fiber intake with mortality may thus to some extent be explained by residual confounding. Our total study population was sufficiently large to detect HRs of approximately 0.85 with 80% power. Therefore, the sample size of our total study population was large enough to detect meaningful associations, but our sample size may have been limited for certain subgroup analyses.

The American Heart Association, and the American Diabetes Association stress the importance of glycemic control in the primary prevention of CVD in patients with diabetes [23], but current guidelines include different recommendations regarding carbohydrate consumption [23], [24]. Although results have to be confirmed in future studies, our data suggest that to improve survival of individuals with diabetes mellitus, nutritional advice should focus on increasing intake of dietary fiber. Our finding that reducing intake of total carbohydrate and sugar may increase survival in normal weight individuals with diabetes mellitus should be subject of further investigation.

## Supporting Information

Figure S1 Forest plot showing country-specific and combined effect estimates for the association between dietary fiber intake and all cause mortality. Adjusted Hazard Ratios (with 95% CI) per SD of daily fiber intake (6.4 g). Age was used as the primary time variable. Models were stratified on sex, and adjusted for CVD-related, diabetes-related, and nutritional risk factors (see Table 2, model M4 for dietary fiber). The overall estimate was based on a random-effect model.
(TIF)

Table S1 Baseline characteristics of the study population, according to lower and upper quartiles of daily nutritional dietary fiber intake, and total carbohydrate (CHO) intake.
(DOC)

Table S2 Baseline characteristics of under- and over-reporters of energy intake.
(DOC)

Table S3 Baseline characteristics of normal and overweight diabetic patients.
(DOC)
