# Supplementary figures and images for: Dietary Fiber, Carbohydrate Quality and Quantity, and Mortality Risk of Individuals with Diabetes Mellitus
Source: PLoS One. 2012 Aug 23;7(8):e43127. doi: 10.1371/journal.pone.0043127 (PMC3426551; doi:10.1371/journal.pone.0043127)

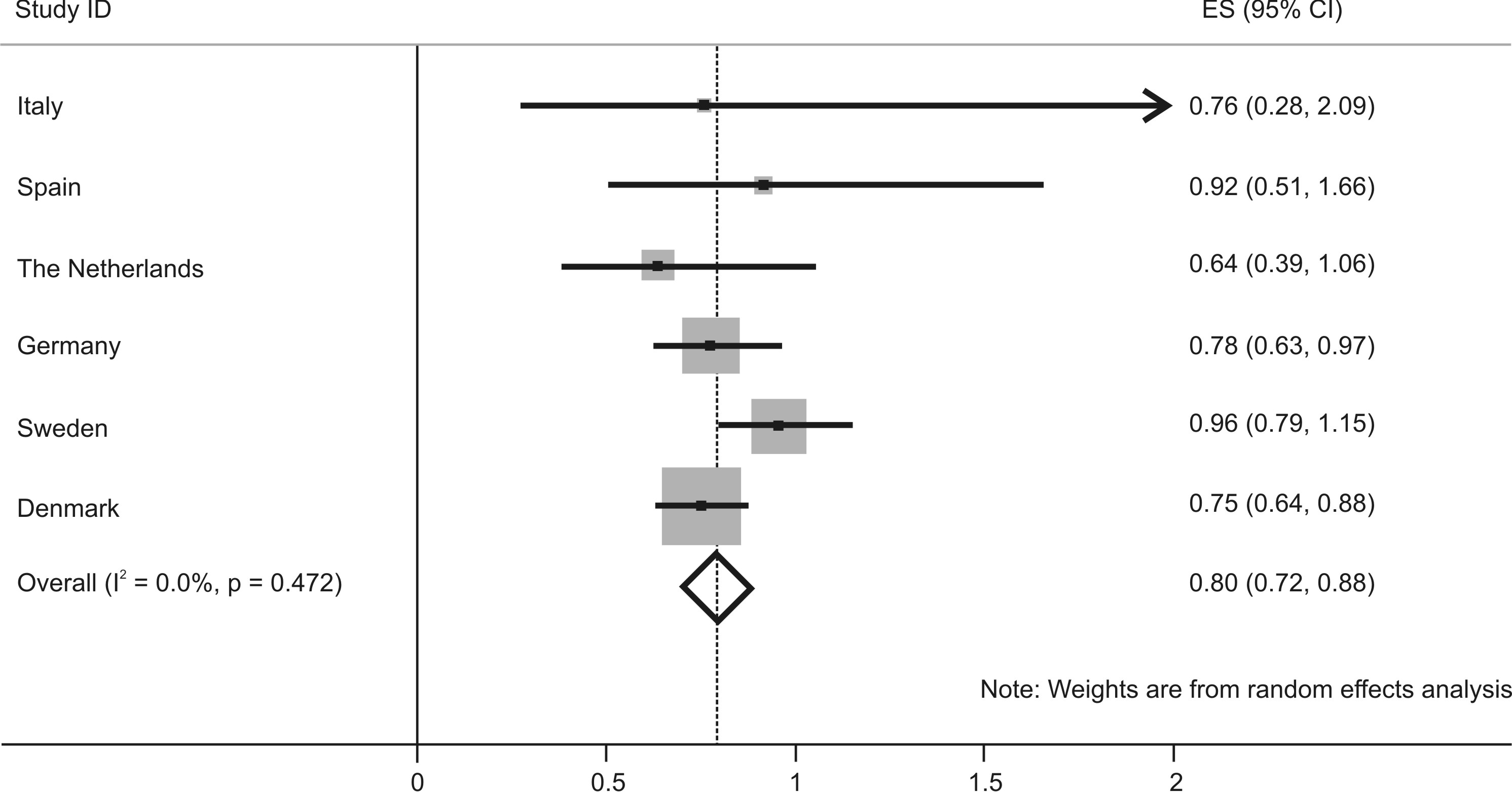

Supplement: Figure S1 — Forest plot showing country-specific and combined effect estimates for the association between dietary fiber intake and all cause mortality. Adjusted Hazard Ratios (with 95% CI) per SD of daily fiber intake (6.4 g). Age was used as the primary time variable. Models were stratified on sex, and adjusted for CVD-related, diabetes-related, and nutritional risk factors (see Table 2, model M4 for dietary fiber). The overall estimate was based on a random-effect model. (TIF) [file pone.0043127.s001.tif]
