# Supplementary material for: Dietary Fiber, Carbohydrate Quality and Quantity, and Mortality Risk of Individuals with Diabetes Mellitus
Source: PLoS One. 2012 Aug 23;7(8):e43127. doi: 10.1371/journal.pone.0043127 (PMC3426551; doi:10.1371/journal.pone.0043127)
Supplement: Table S1 — Baseline characteristics of the study population, according to lower and upper quartiles of daily nutritional dietary fiber intake, and total carbohydrate (CHO) intake. (DOC) [file pone.0043127.s002.doc]

|  | | **Fiber Q1** | **Q4** | **CHO Q1** | **Q4** |  |
| --- | --- | --- | --- | --- | --- | --- |
|  | |  |
| N | | 1548 | 1548 | 1548 | 1548 |  |
| Male sex (n, %) | | 982(63.4) | 749 (48.4) | 1017 (65.7) | 722 (46.6) |  |
| Age (yrs) | | 56.9 ± 7.0* | 57.7 ± 6.0 | 56.8 ± 6.4 | 57.4 ± 6.9 |  |
| BMI (kg/m^2^) | | 29.0 ± 4.8 | 28.4 ± 5.1 | 28.9 ± 4.7 | 28.5 ± 5.0 |  |
| WHR | | 0.94 ± 0.09 | 0.91 ± 0.09 | 0.94 ± 0.09 | 0.91 ± 0.09 |  |
| Physical Activity (%) | |  |  |  |  |  |
|  | Inactive | 35.2 | 25.6 | 30.5 | 31.5 |  |
|  | Mod Inactive | 31.4 | 31.2 | 31.0 | 32.9 |  |
|  | Mod Active | 19.4 | 21.8 | 21.4 | 19.8 |  |
|  | Active | 14.0 | 21.4 | 17.1 | 15.8 |  |
| Education (%) | |  |  |  |  |  |
|  | Low | 44.1 | 47.2 | 44.9 | 46.0 |  |
|  | Middle | 38.3 | 37.3 | 36.6 | 38.3 |  |
|  | High | 17.6 | 15.6 | 18.5 | 15.7 |  |
| Smoking (%) | |  |  |  |  |  |
|  | Never | 33.6 | 43.6 | 32.3 | 45.7 |  |
|  | Former | 36.5 | 35.3 | 35.4 | 34.7 |  |
|  | Current | 30.0 | 21.0 | 32.3 | 19.6 |  |
| HbA1c (% of total hemoglobin) | | 8.1 ± 1.9 | 8.1 ± 2.0 | 7.9 ± 1.7 | 8.1 ± 2.1 |  |
| Menopausal status (% post) | | 68.7 | 83.0 | 71.9 | 79.4 |  |
| HRT use (%) | | 11.9 | 16.3 | 14.4 | 14.6 |  |
| Duration of diabetes (yrs) | | 4.3 (1.8-10.1)** | 4.3 (1.9-7.6) | 4.6 (2.2-10.3) | 3.9 (1.5-9.1) |  |
| Insulin use (%) | | 16.5 | 28.9 | 22.4 | 22.0 |  |
| Nutrients (daily intake)† | |  |  |  |  |  |
|  | Total Energy (kcal) | 2091 ± 643 | 2069 ± 616 | 2095 ± 675 | 2094 ± 678 |  |
|  | Carbohydrate (g) | 193.8 ± 37.5 | 228.5 ± 31.2 | 167.0 ± 18.1 | 256.4 ± 19.1 |  |
|  | Polyunsaturated Fat (g) | 12.6 ± 4.7 | 13.4 ± 4.7 | 14.4 ± 5.7 | 11.6 ± 3.4 |  |
|  | Monounsaturated Fat (g) | 29.8 ± 7.3 | 25.4 ± 7.2 | 32.9 ± 8.5 | 23.2 ± 5.0 |  |
|  | Saturated Fat (g) | 31.4 ± 8.4 | 25.8 ± 6.2 | 31.5 ± 8.5 | 25.2 ± 5.4 |  |
|  | Fiber (g) | 16.1 ± 2.5 | 32.1 ± 4.5 | 20.0 ± 5.3 | 26.5 ± 7.0 |  |
|  | Alcohol (g) | 11.7 (1.9-38.3) | 3.2 (0.4-12.3) | 17.1 (3.6-44.0) | 2.0 (0.2-7.8) |  |
|  | Vitamin C (mg) | 89.0 ± 44.5 | 139.4 ± 68.0 | 99.2 ± 50.9 | 133.5 ± 64.9 |  |

Table S1. Baseline characteristics of the study population, according to lower and upper quartiles of daily nutritional dietary fiber intake, and total carbohydrate (CHO) intake

*Mean ± SD (all such values); **Median (IQR; all such values); †nutritional variables were adjusted for total energy intake, except alcohol and energy. Q1 and Q4, lower and upper quartile. CHO = total carbohydrate; BMI = body mass index; WHR= waist-to-hip ratio; HRT = hormone replacement therapy
