# Supplementary material for: Dietary Fiber, Carbohydrate Quality and Quantity, and Mortality Risk of Individuals with Diabetes Mellitus
Source: PLoS One. 2012 Aug 23;7(8):e43127. doi: 10.1371/journal.pone.0043127 (PMC3426551; doi:10.1371/journal.pone.0043127)
Supplement: Table S2 — Baseline characteristics of under- and over-reporters of energy intake. (DOC) [file pone.0043127.s003.doc]

Table S2. Baseline Characteristics of under- and over-reporters of energy intake

|  | | **Under-reporters** | **Over-reporters** |
| --- | --- | --- | --- |
|  | |
| N | | 2301 | 53 |
| Male sex (n, %) | | 1183 (51.4) | 33 (62.3) |
| Glycemic Load (g/d) | | 115.9 ± 21.7* | 121.8 ± 26.7 |
| Glycemic Index | | 55.2 ± 3.9 | 54.2 ± 4.7 |
| Age (yrs) | | 57.2 ± 6.3 | 56.5 ± 7.6 |
| BMI (kg/m^2^) | | 30.3 ± 5.0 | 27.3 ± 5.2 |
| WHR | | 0.93 ± 0.09 | 0.93 ± 0.09 |
| Physical Activity (%) | |  |  |
|  | Inactive | 32.8 | 25.0 |
|  | Mod Inactive | 32.1 | 34.6 |
|  | Mod Active | 20.5 | 19.2 |
|  | Active | 14.5 | 21.2 |
| Education (%) | |  |  |
|  | Low | 48.4 | 41.5 |
|  | Middle | 37.4 | 43.4 |
|  | High | 14.2 | 15.1 |
| Smoking (%) | |  |  |
|  | Never | 40.8 | 32.1 |
|  | Former | 37.1 | 22.6 |
|  | Current | 22.1 | 45.3 |
| Systolic blood pressure (mm Hg) | | 145.8 ± 20.6 | 146.2 ± 21.4 |
| Diastolic blood pressure (mm Hg) | | 86.5 ± 11.1 | 86.7 ± 10.3 |
| Hypertension (%) | | 58.0 | 61.7 |
| Hypercholesterolemia (%) | | 47.0 | 31.8 |
| HbA1c (% of total hemoglobin) | | 8.1 ± 1.9 | 8.0 ± 2.3 |
| Menopausal status (% post) | | 78.4 | 90.0 |
| OC use (%) | | 1.5 | 0.0 |
| HRT use (%) | | 13.5 | 5.0 |
| Age at diabetes diagnosis (yrs) | | 49.8 ± 9.4 | 50.1 ± 10.0 |
| Duration of diabetes (yrs) | | 4.6 (1.7-10.1)** | 4.4 (2.4-9.7) |
| Insulin use (%) | | 21.1 | 18.9 |
| Use of glucose-lowering drugs (%) | | 82.3 | 83.3 |
| Nutrients (daily intake)† | |  |  |
|  | Total Energy (kcal) | 1547 ± 315 | 3962 ± 672 |
|  | Carbohydrate (g) | 209.6 ± 35.3 | 224.6 ± 42.3 |
|  | Sugar (g) | 82.4 ± 29.7 | 102.5 ± 50.8 |
|  | Starch (g) | 120.9 ± 31.0 | 119.1 ± 43.2 |
|  | Protein (g) | 89.1 ± 16.7 | 89.0 ± 18.5 |
|  | Total Fat (g) | 76.2 ± 13.9 | 77.6 ± 13.5 |
|  | Polyunsaturated Fat (g) | 13.2 ± 4.5 | 13.3 ± 5.9 |
|  | Monounsaturated Fat (g) | 28.0 ± 7.7 | 28.5 ± 6.7 |
|  | Saturated Fat (g) | 28.9 ± 7.3 | 29.9 ± 7.5 |
|  | Fiber (g) | 23.1 ± 6.3 | 23.8 ± 7.8 |
|  | Alcohol (g) | 2.9 (0.2-11.7) | 13.2 (0.2-34.7) |
|  | Vitamin C (mg) | 111.3 ± 54.8 | 145.7 ± 88.4 |

*Mean ± SD (all such values); **Median (IQR; all such values); energy under- and over- reporters were defined as energy intake compared to basal metabolic rate of <1.14 and > 2.40, respectively; †nutritional variables were adjusted for total energy intake, except alcohol and energy. Note the low number of energy over-reporters. There were 283 death cases (185 men) among energy under-reporters, 121 (83 men) died of CVD, 66 (41 men) died of cancer, and 41 (23 men) died of other known causes. There were 10 death cases (6 men) among energy over-reporters, 4 (3 men) died of CVD, 2(2 men) died of cancer, and 2 (1 man) died of other known causes. BMI = body mass index; WHR= waist-to-hip ratio; OC= oral contraceptives; HRT = hormone replacement therapy
