# Supplementary material for: Dietary Fiber, Carbohydrate Quality and Quantity, and Mortality Risk of Individuals with Diabetes Mellitus
Source: PLoS One. 2012 Aug 23;7(8):e43127. doi: 10.1371/journal.pone.0043127 (PMC3426551; doi:10.1371/journal.pone.0043127)
Supplement: Table S3 — Baseline characteristics of normal and overweight diabetic patients. (DOC) [file pone.0043127.s004.doc]

Table S3. Baseline Characteristics of normal and overweight diabetic patients

|  | | **BMI ≤ 25 kg/m^2^** | **BMI > 25 kg/m^2^** |
| --- | --- | --- | --- |
|  | |
| N (n, %) | | 1353 (21.9) | 4839 (78.1) |
| Energy under-reporters (n, %) | | 288 (21.3) | 2013 (41.6) |
| Energy over-reporters (n, %) | | 18 (1.3) | 35 (0.7) |
| Male sex (n, %) | | 706 (52.2) | 2649 (54.7) |
| Glycemic Load (g/d) | | 119.7 ± 22.1* | 116.2 ± 21.8 |
| Glycemic Index | | 55.5 ± 4.1 | 55.1 ± 3.9 |
| Age (yrs) | | 56.6 ± 7.3 | 57.7 ± 6.4 |
| BMI (kg/m^2^) | | 22.9 ± 1.7 | 30.5 ± 4.2 |
| WHR | | 0.87 ± 0.08 | 0.94 ± 0.08 |
| Physical Activity (%) | |  |  |
|  | Inactive | 26.4 | 31.7 |
|  | Mod Inactive | 33.4 | 32.2 |
|  | Mod Active | 20.9 | 19.8 |
|  | Active | 19.3 | 16.4 |
| Education (%) | |  |  |
|  | Low | 34.9 | 48.8 |
|  | Middle | 44.9 | 35.9 |
|  | High | 20.3 | 15.3 |
| Smoking (%) | |  |  |
|  | Never | 38.4 | 39.5 |
|  | Former | 30.5 | 37.2 |
|  | Current | 31.2 | 23.4 |
| Systolic blood pressure (mm Hg) | | 140.3 ± 22.1 | 146.7 ± 20.5 |
| Diastolic blood pressure (mm Hg) | | 81.2 ± 10.3 | 86.6 ± 10.9 |
| Hypertension (%) | | 33.8 | 55.4 |
| Hypercholesterolemia (%) | | 31.3 | 45.4 |
| HbA1c (% of total hemoglobin) | | 8.0 ± 1.9 | 8.1 ± 1.9 |
| Menopausal status (% post) | | 74.0 | 79.1 |
| OC use (%) | | 3.3 | 1.2 |
| HRT use (%) | | 18.5 | 12.4 |
| Age at diabetes diagnosis (yrs) | | 47.9 ± 11.5 | 50.7 ± 9.2 |
| Duration of diabetes (yrs) | | 5.2 (2.2-10.9)** | 4.2 (1.7-9.2) |
| Insulin use (%) | | 38.1 | 17.9 |
| Use of glucose-lowering drugs (%) | | 69.3 | 85.4 |
| Nutrients (daily intake)† | |  |  |
|  | Total Energy (kcal) | 2088 ± 589 | 2070 ± 653 |
|  | Carbohydrate (g) | 215.1 ± 34.8 | 210.5 ± 35.5 |
|  | Sugar (g) | 82.0 ± 29.9 | 85.2 ± 31.2 |
|  | Starch (g) | 128.6 ± 32.1 | 119.9 ± 31.2 |
|  | Protein (g) | 88.5 ± 15.5 | 89.5 ± 16.1 |
|  | Total Fat (g) | 75.7 ± 13.1 | 76.5 ± 13.6 |
|  | Polyunsaturated Fat (g) | 12.9 ± 4.1 | 13.3 ± 4.7 |
|  | Monounsaturated Fat (g) | 27.4 ± 7.0 | 28.2 ± 7.4 |
|  | Saturated Fat (g) | 29.3 ± 7.3 | 28.8 ± 7.3 |
|  | Fiber (g) | 24.6 ± 6.8 | 23.2 ± 6.3 |
|  | Alcohol (g) | 6.8 (0.8-20.4) | 5.4 (0.5-20.8) |
|  | Vitamin C (mg) | 110.8 ± 50.3 | 115.2 ± 57.0 |

*Mean ± SD (all such values); **Median (IQR; all such values); †nutritional variables were adjusted for total energy intake, except alcohol and energy. There were 183 death cases among normal weight and 608 among overweight subjects, 70(236) died of CVD, 42 (121) died of cancer, and 32 (86) died of other known causes. BMI = body mass index; WHR= waist-to-hip ratio; OC= oral contraceptives; HRT = hormone replacement therapy
